# Maternal psychological distress in primary care and association with child behavioural outcomes at age three

**DOI:** 10.1007/s00787-015-0777-2

**Published:** 2015-09-28

**Authors:** Stephanie L. Prady, Kate E. Pickett, Tim Croudace, Dan Mason, Emily S. Petherick, Rosie R.C. McEachan, Simon Gilbody, John Wright

**Affiliations:** Department of Health Sciences, University of York, York, YO10 5DD UK; School of Nursing and Health Sciences, University of Dundee, Dundee, DD1 4HN UK; Social Dimensions of Health Institute, University of Dundee, Dundee, DD1 4HN UK; Bradford Institute for Health Research, Bradford Royal Infirmary, Duckworth Lane, Bradford, BD9 6RJ UK; Hull York Medical School, University of York, York, YO10 5DD UK

**Keywords:** Health inequality, Maternal mental health, Anxiety, Depression, Strengths and Difficulties Questionnaire, Latent class methods

## Abstract

Observational studies indicate children whose mothers have poor mental health are at increased risk of socio-emotional behavioural difficulties, but it is unknown whether these outcomes vary by the mothers’ mental health recognition and treatment status. To examine this question, we analysed linked longitudinal primary care and research data from 1078 women enrolled in the Born in Bradford cohort. A latent class analysis of treatment status and self-reported distress broadly categorised women as (a) not having a common mental disorder (CMD) that persisted through pregnancy and the first 2 years after delivery (*N* = 756, 70.1 %), (b) treated for CMD (*N* = 67, 6.2 %), or (c) untreated (*N* = 255, 23.7 %). Compared to children of mothers without CMD, 3-year-old children with mothers classified as having untreated CMD had higher standardised factor scores on the Strengths and Difficulties Questionnaire (*d* = 0.32), as did children with mothers classified as having treated CMD (*d* = 0.27). Results were only slightly attenuated in adjusted analyses. Children of mothers with CMD may be at risk for socio-emotional and behavioural difficulties. The development of effective treatments for CMD needs to be balanced by greater attempts to identify and treat women.

**Electronic supplementary material:**

The online version of this article (doi:10.1007/s00787-015-0777-2) contains supplementary material, which is available to authorized users.

## Background

Research evidence points to an association between poor parental mental health and increased socio-emotional and behavioural disturbance in their children [1–7]. Much of this evidence base comes from observational research studies which classify mothers’ and sometimes fathers’ mental health problems using scores derived from screening measures or diagnostic interviews by professionals. These studies quantify the scale of the problem and the nature of the association but their contribution is limited because they only rarely account for impact of treatment received by the parent from health services. In clinical practice, up to half of women with common mental health disorders (CMD, anxiety and depression) remain undiagnosed, i.e. are not identified and, therefore, are unlikely to be offered psychiatric treatment for their disorder or receive parenting or attachment support to mitigate potentially harmful outcomes for their child [8–10].

A small evidence base indicates that maternal psychotherapy treatment may improve outcomes in mothers and children [11], and to date few differences in child outcomes have been noted by the type of treatment during pregnancy [12–14]. The evidence base, however, has only limited characterisation of outcomes in families where a women’s distress remains unidentified to the health service. Consequently, we not know whether or not children of women with unidentified, and therefore untreated, CMD have an elevated risk of problems and thus the health and social problems stemming from unidentified CMD cannot be quantified.

In a previous analysis, we used linked primary care and research data from a city-based birth cohort (Born in Bradford, BiB) to estimate that up to 50 % of women with CMD during pregnancy were potentially unidentified (not detected) in primary care [10]. Here, we extend that work through analysis of linked data from a sub-set of women in BiB who were asked to complete further self-reported psychological distress measures in the postnatal period and also rated their child’s socio-emotional behavioural problems at age three. Our aim is to describe variation in socio-emotional behavioural problems in 3-year-old offspring of women who had persistent untreated CMD in the maternal period, compared to offspring of women without CMD and those who were treated in primary care for CMD.

## Methods

### Sample

The data for this analysis come from women recruited to the Born in Bradford (BiB) cohort, a longitudinal community study aiming to examine environmental, psychological and genetic factors and their impact on maternal and child health [15, 16]. Bradford is a city in the North of England with high levels of socio-economic deprivation and ethnic diversity. Women were consecutively approached for recruitment while waiting for their glucose tolerance test, a routine procedure offered to all pregnant women registered at the Bradford Royal Infirmary, at 26–28 weeks gestation. More than 80 % of women consented to take part and the cohort recruited 12,450 women antenatally between 2007 and 2010, resulting in 13,857 live births. We analysed a sub-set of the full BiB cohort (BiB1000) as only these women contributed longitudinal self-reported data. Women in BiB1000 were consecutive enrollees between the dates August 2008 and March 2009 who completed the recruitment questionnaire and consented to repeat visits at 6, 12, 18, 24 and 36 months after delivery [17]. Out of 1917 eligible pregnancies, 1735 mothers agreed to take part in the BiB1000 study. Ethical approval for the data collection was granted on April 1, 2008 by Bradford Research Ethics Committee (Ref 07/H1302/112).

### Exclusions

We excluded 657 of these 1735 women (37.9 %) from our analysis, leaving an analytic sample of *N* = 1078. Exclusions could be for more than one reason. To minimise potential unknown missing data bias caused by some women having an incomplete GP record because they moved to a practice not using the computer system that provided primary care data, we used NHS tracing files to exclude women who relocated from Bradford between recruitment and up to 2 years after the delivery (*N* = 238). We excluded women whose research data could not be linked with the GP record (*N* = 127), those with codes or prescriptions indicating the possibility of severe mental illness (*N* = 113) and those without a questionnaire (SDQ) when the child was aged three (*N* = 533). We only analysed data on the first-born of the 25 sets of twins.

### Outcome measure: Strengths and Difficulties Questionnaire

At the 36th month interview the children’s mothers were administered the 25-item Strengths and Difficulties Questionnaire (SDQ) [18]. In non-clinical samples the SDQ may be expected to assess early manifestations of two theorised dimensions of children’s problem behaviour (usually applied to older children and adolescents); internalising and externalising, which are combined into a Total Difficulties score, and a positive, pro-social scale. Each item is rated on a scale from 0 (best) to 2 (worst) with five positively worded problem items having reversed scales. A psychometric analysis of the SDQ in this sample revealed less support for the underlying theorised structure of internalising and externalising dimensions [19]. Factor analyses in other pre-school samples have also found it hard to confirm the theorised structures [20, 21]. The latent structure variation we found may have been a consequence of the very young age of the children, or related to heterogeneity in this multi-ethnic sample. We found, as have others [22, 23], that a bifactor model, which isolates a general factor or dominant dimension of variation from the internalising, externalising and positively worded questions, fitted the data from this sample well. In this analysis we used standardised scores from this factor, generated after fitting the bifactor model to the whole sample. These scores are estimates for the loading on the Total Difficulties factor after partitioning out the error variance. This procedure computed a single score on the major dimension (latent bifactor) for each child from the 20 problem behaviour items indicating a child’s position relative to others in the sample. The correlation between the standardised factor scores generated this way and the Total Difficulties scores computed in the standard way using a simple sum score was high at 0.90.

As a sensitivity analysis for the factor score method of assessing child behavioural problems we also used the single-item ‘Perceived Difficulties’ question in the impact supplement of the SDQ which asks; “Overall, do you think that your child has difficulties in one or more of the following areas: emotions, concentration, behaviour or being able to get on with other people?”. The response scale is ‘severe’, ‘serious’, ‘minor’ and ‘none’. The distribution of responses in our sample was 2 % rated as having ‘severe’ or ‘serious’ difficulties, 10 % with minor difficulties, and 88 % no difficulties. The proportion of children with difficulties appears low compared to other community samples of older children (mean age ~10). Around 5 % of parents in UK and German studies reported that their child had serious or severe difficulties and ~30 % of children were rated as having only minor problems in the UK study [24, 25]. As it was unclear whether this was an age effect, we classified the children as having difficulties if their mother rated them as having either minor, serious or severe difficulties (12 %).

### Predictor: maternal mental health

The maternal mental health predictor was derived in three stages: dichotomised self-reported data; treatment status derived from the medical record; latent class analysis using both stages one and two.

#### Classification of psychological distress from self-reported measures

Data from self-reported psychological distress measures (expected to capture variation in non-psychotic psychiatric risk) were collected at various points by the research team; the GHQ-28 during pregnancy, at 6 and 18 months after the baby’s birth, and the Kessler-6 at 12 and 24 months postnatally [26, 27]. For the GHQ-28 we performed a simple imputation of zero for questionnaires with fewer than four items missing, which numbered only a handful of cases in each assessment period. We considered the questionnaire missing if the GHQ-28 was missing between 5 and 28 items or if the Kessler-6 was missing any items. The ethnic diversity of women in BiB presents a challenge for comparing scores from standardised measures, as questions cannot be assumed to have equal relationships to psychopathology across cultures [28, 29]. To minimise any effect we used non-parametric determinations, considering women with scores at and over the 75th centile of their within-ethno-language group to be psychologically distressed [30]. The four groups we used to classify scores were (1) White British with administration of the recruitment questionnaire in English (*N* = 396), (2) Pakistani with administration in English (*N* = 329), (3) all other administrations in English (*N* = 123), and (4) administrations in a language other than English (nearly all of these were Pakistani women using Urdu, *N* = 230). Where language was missing we assumed English (*N* = 6), where the woman’s ethnic group was missing we assumed ‘Other ethnicity’ (*N* = 2). We selected the 75th centile as a non-conservative threshold for CMD, as best estimates of point prevalence of depression are around 10 % during pregnancy and 7–13 % during the first year postpartum, with a period prevalence of up to 20 % during the first 3 months postpartum [31].

We grouped the five self-reported binary measures to approximate the three time periods classified in the medical record (Table 1).

**Table 1:** Classification of risk by self-reported measures

Classification	Self-report at risk pre-birth	Self-report at risk year 1		Self-report at risk year 2	
Self-reported measure	GHQ-28	GHQ-28	Kessler-6	GHQ-28	Kessler-6
Timing	Pregnancy	6 months	12 months	18 months	24 months
Considered ‘at risk’ if	Score is ≥75th centile^a^	At least one score is ≥75th centile^a^		At least one score is ≥75th centile^a^	
Missing if	Score is missing	Both scores are missing		Both scores are missing	

^a^Centiles were calculated within ethno-language group to minimise variation caused by potential measurement inconsistencies

#### Classification of ‘treated CMD’ from GP records

Bradford’s primary care practices use SystmOne clinical software (©TPP) in which clinical and administrative terms are classified by Read codes, and prescriptions listed by the British National Formulary. SystmOne electronic primary care records (‘GP records’) were matched to BiB research records by a third party data provider using NHS number up to February 2013. We compiled lists of Read codes [32] relevant to the treatment, or referral for treatment, of CMD. We searched the GP records of participants for these Read codes, and for drugs used to treat CMD, during the study period. We had no access to free-text notes and referral letters. A list of Read codes and medications are in supplementary appendix table S1.

We coded for the presence or absence of a pharmacologic or non-pharmacologic treatment for CMD in the GP record over three periods; (1) the 6-month preconception period and pregnancy ‘pre-birth’, (2) the first postnatal year, and (3) the second postnatal year.

### Latent class analysis

We undertook latent class analysis (LCA) modelling to identify groups of women with similar patterns of mental health/mental distress and treatment status across the study period. We modelled six binary variables, three relating to treatment from the medical record and three derived from self-reported measures. We used MPlus version 7.2 to estimate latent class models using full information maximum likelihood (FIML) to account for missing data, assuming that mental health screening measures were missing completely at random (MCAR). Missing screening measure data ranged from 0.4 % (*N* = 4) in pregnancy to 3.6 % (*N* = 39) in the first postnatal year (both 6 and 12 months data missing). Missingness was not predicted by correlations between the mental health variables and the SDQ (all correlations for missing data <0.075). The optimal number of latent classes was derived by assessing; (1) the model with the lowest values of two information criteria—Akaike’s information criterion (AIC) and Bayesian information criterion (BIC), (2) the results of the Vuong–Lo–Mendell–Rubin-adjusted likelihood ratio test, and (3) considerations of parsimony with no trivial classifications (i.e. very small or unusual groupings). A Pearson test statistic >30 for any bivariate pair was considered as an indication that there was a likely violation of the assumption that variables used in the model were independent of one another [33], conditional on the latent class model.

### Covariates

The following variables were used as covariates in regression analyses. We entered the mother’s ethnicity as categorised above, the baby’s sex as noted in the electronic maternity system and, as a marker of socio-economic deprivation, the mother’s responses to the 12-item Family Resources Survey (FRS) Adult Deprivation Questions [34] when recruited. The score was derived from a count of the number of items which the mother indicated they could not afford (I/we would like this but cannot afford it at this moment). All responses in the category ‘I/we do not want/need this at this moment’ together with responses of ‘Do not know’ were classified with responses indicating the items could be afforded. If mothers did not provide a response to all the material deprivation questions her response was set to missing. We used the FRS rather than other markers of socio-economic status such as income or area-based deprivation because a substantial number of mothers in BiB did not know or report their income and there is little variation in the small area-based deprivation score between different areas of Bradford, with over 85 % of mothers in this sample living in the 40 % most deprived areas nationally.

### Statistics

We calculated means, effect sizes (Cohen’s *d* and point-biserial *r*) and 95 % CI of standardised SDQ factor scores by latent classification of CMD status. We modelled the effect of latent classification of CMD on standardised SDQ factor scores in three sequential steps. In the first, we adjusted for child sex only, in the second model we also adjusted for mother’s ethnic group to observe the effect of any ethnicity-related variation in scoring children’s behaviour. To establish whether differences were grossly attributable to variation in socio-economic status we also adjusted for the number of items lacked on the FRS in the third model. We used linear regression with robust standard errors; reporting beta coefficients and 95 % CI. We used Poisson regression to estimate similar models for the Perceived Difficulties question, reporting relative risk ratios (RRR) and their 95 % CI. Stata 13 [35] was used for all analyses except the latent classification, where we used Mplus 7.2 [36].

## Results

### Participation

We analysed data on 1078 women. Of these, the numbers participating in each sweep were pregnancy *N* = 1078 (100 %), 6 months postnatal *N* = 918 (85.2 %), 12 months *N* = 946 (87.8 %), 18 months *N* = 983 (91.2 %), 24 months *N* = 991 (91.9 %), and 36 months *N* = 1078 (100 %). Sixty-seven percent (*N* = 726) participated at every sweep.

### Participants

Women included in the analysis were less likely to be White British, more likely to have used English for the baseline questionnaire, be on average a year older and married than women excluded (Table 2). There was little evidence of differences in the socio-economic status, self-reported psychological distress, CMD treatment in GP notes, or perceived difficulties on the SDQ between the included and excluded sample. No marked differences in the characteristics of the women excluded for different reasons were evident (Supplementary online appendix Table S2).

**Table 2:** Descriptive characteristics of the included and excluded sample

	Analysed	Excluded	Analysed vs. excluded *χ* ^2^ (*df*), *P*
*N*	1078	657	
Ethnic group (language), *N* (%)			
White British	396 (36.7)	264 (40.1)	15.1 (3), 0.002
Pakistani (English)	329 (30.5)	184 (28.0)	
Other (English)	123 (11.4)	105 (16.0)	
Any (not English)	230 (21.3)	104 (15.8)	
Migration history			
Born in the UK	664 (61.6)	419 (63.8)	1.1 (2), 0.57
Migrated to UK before age 16	81 (7.5)	43 (6.5)	
Migrated to UK on or after age 16	327 (30.3)	190 (28.9)	
Missing	6 (0.6)	5 (0.8)	
Age at enrolment, mean (SD)	27.6 (5.7)	26.5 (5.6)	^b^ *t* = 3.9, *P* < 0.001
Relationship status at recruitment *N* (%)			
Married and living together	760 (70.5)	415 (63.2)	14.1 (2), 0.001
Cohabiting	181 (16.8)	120 (18.3)	
Not living with a partner	133 (12.3)	121 (18.4)	
Missing	4 (0.4)	1 (0.2)	
IMD national rank quintile			
Most deprived	718 (66.6)	456 (69.4)	3.4 (4), 0.49
2	206 (19.1)	109 (16.6)	
3	112 (10.4)	70 (10.7)	
4	27 (2.5)	11 (1.7)	
Least deprived	15 (1.4)	11 (1.7)	
Number of items lacked on the Family Resources Survey at recruitment			
None	461 (42.8)	280 (42.6)	1.3 (3), 0.74
1–2	298 (27.6)	170 (25.9)	
3–4	165 (15.3)	98 (14.9)	
5+	145 (13.5)	99 (15.1)	
Missing	9 (0.8)	10 (1.5)	
≥75th centile of self-reported distress measure, *N* (%)^a,c^			
GHQ-28 during pregnancy	302 (28.1)	174 (26.8)	0.35 (1), 0.56
Missing	4 (0.4)	8 (1.2)	
GHQ-28 at 6 months	257 (28.7)	120 (30.8)	0.57 (1), 0.45
Missing	182 (16.9)	267 (40.6)	
Kessler-6 at 12 months	258 (27.4)	113 (32.5)	3.2 (1), 0.07
Missing	136 (12.6)	309 (47.0)	
GHQ-28 at 18 months	251 (25.8)	89 (30.4)	2.3 (1), 0.13
Missing	107 (9.9)	364 (55.4)	
Kessler-6 at 24 months	248 (25.2)	70 (31.3)	3.4 (1), 0.06
Missing	94 (8.7)	433 (65.9)	
Has CMD treatment Read codes/prescriptions, *N* (%)			
*N*	1078	322	
Pre-birth	79 (7.3)	31 (9.7)	1.8 (1), 0.18
In first postnatal year	118 (11.0)	40 (12.5)	0.54 (1), 0.46
In second postnatal year	139 (12.9)	47 (14.6)	0.62 (1), 0.43
SDQ Perceived Difficulties question, *N* (%)^a^			
*N*	1078	335	
Severe, serious or minor	137 (12.7)	20 (16.8)	1.5 (1), 0.22
Missing	0	216 (64.5)	

Excluded: did not have linked medical records, moved from Bradford, did not participate in any postnatal sweeps, missing SDQ

*IMD* index of multiple deprivation, *GHQ* General Health Questionnaire, *SDQ* Strengths and Difficulties Questionnaire

^a^Percentage of non-missing scores

^b^ *t* test

^c^Centiles presented in this table were calculated using the whole BiB1000 sample (*N* = 1735)

### CMD treatment and psychological distress

Just over 7 % of women had a pre-birth marker of CMD treatment in their record; 11 and 13 % in each subsequent postnatal year (Table 3).

**Table 3:** CMD treatment and psychological distress

	Pre-birth			Year 1 postnatal			Year 2 postnatal		
	*N*	%	95 % CI	*N*	%	95 % CI	*N*	%	95 % CI
Treated for CMD	79	7.3	5.9, 9.0	118	11.0	9.2, 13.0	139	12.9	11.0, 15.0
‘At risk’ on SR screening measure(s)	297	27.7	25.0, 30.4	436^a^	42.0	39.0, 45.0	435^a^	41.2	38.2, 44.2
Missing	4	0.4		39^b^	3.6		21^b^	2.0	

*SR* self-report

^a^Either self-report measure ≥75th centile

^b^Both self-report risk measures for that period missing

### Latent class analysis of maternal mental health

The bootstrapped LR test returned a non-significant result when comparing the five-class solution to the four-class solution, (Supplementary online appendix Table S3); however, we chose the more parsimonious three-class solution as our preferred model. In the four-class solution there was a small group (4 %) of potentially untreated women with varying probabilities of high SR scores over time. We chose not to separate this small group from the larger three classes. In addition, the BIC took its lowest value for the three-class solution. All bivariate Pearson test statistics were <0.6, indicating the model did not violate the assumption of local independence.

**Fig. 1:**
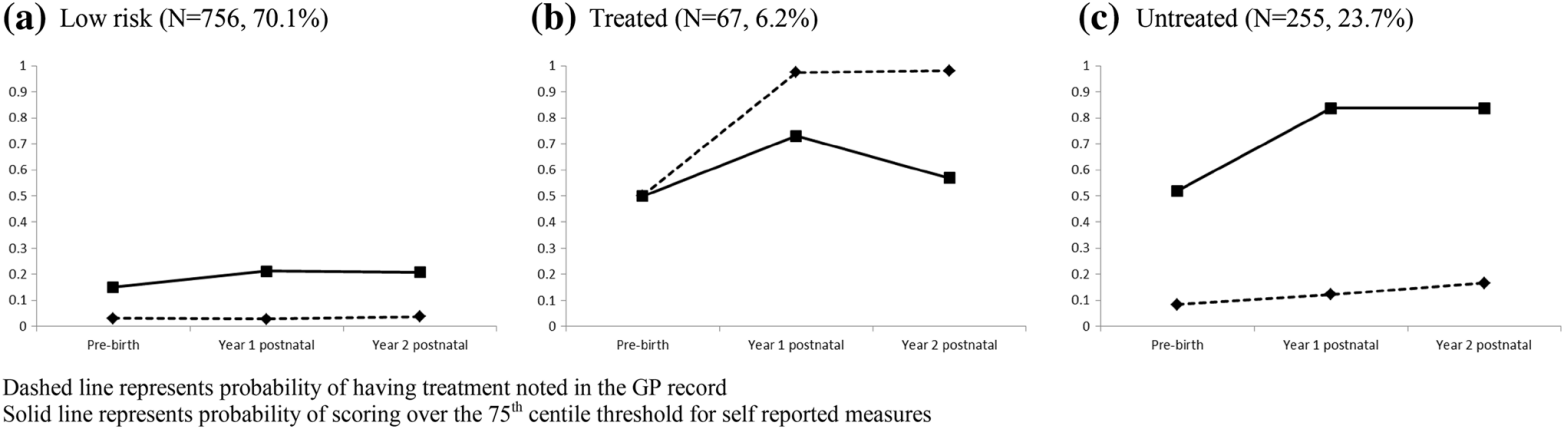
Latent classification of maternal mental health. *Dashed line* probability of having treatment noted in the GP record. *Solid line* probability of scoring at or over the 75th centile threshold for self-reported measures

The LCA classification groups were characterised as follows: (a) women unlikely to have CMD (low probability of treatment and low probability of high self-reported scores) comprising 70.1 % of the sample; (b) ‘Treated’ CMD by the GP (high probability of treatment and high probability of high self-reported scores) comprising 6.2 % of the sample; and (c) potentially ‘untreated’ persistent CMD (low probability of treatment and high probability of high self-reported scores) (23.7 %) (Fig. 1).

### Child behaviour difficulties

One hundred and thirty-seven children (12.7, 95 % CI; 10.8, 14.8) were classed as having ‘severe’, ‘serious’ or ‘minor’ difficulties on the Perceived Difficulties question of the SDQ.

### Associations between latent classification of maternal mental health and SDQ outcomes

**Fig. 2:**
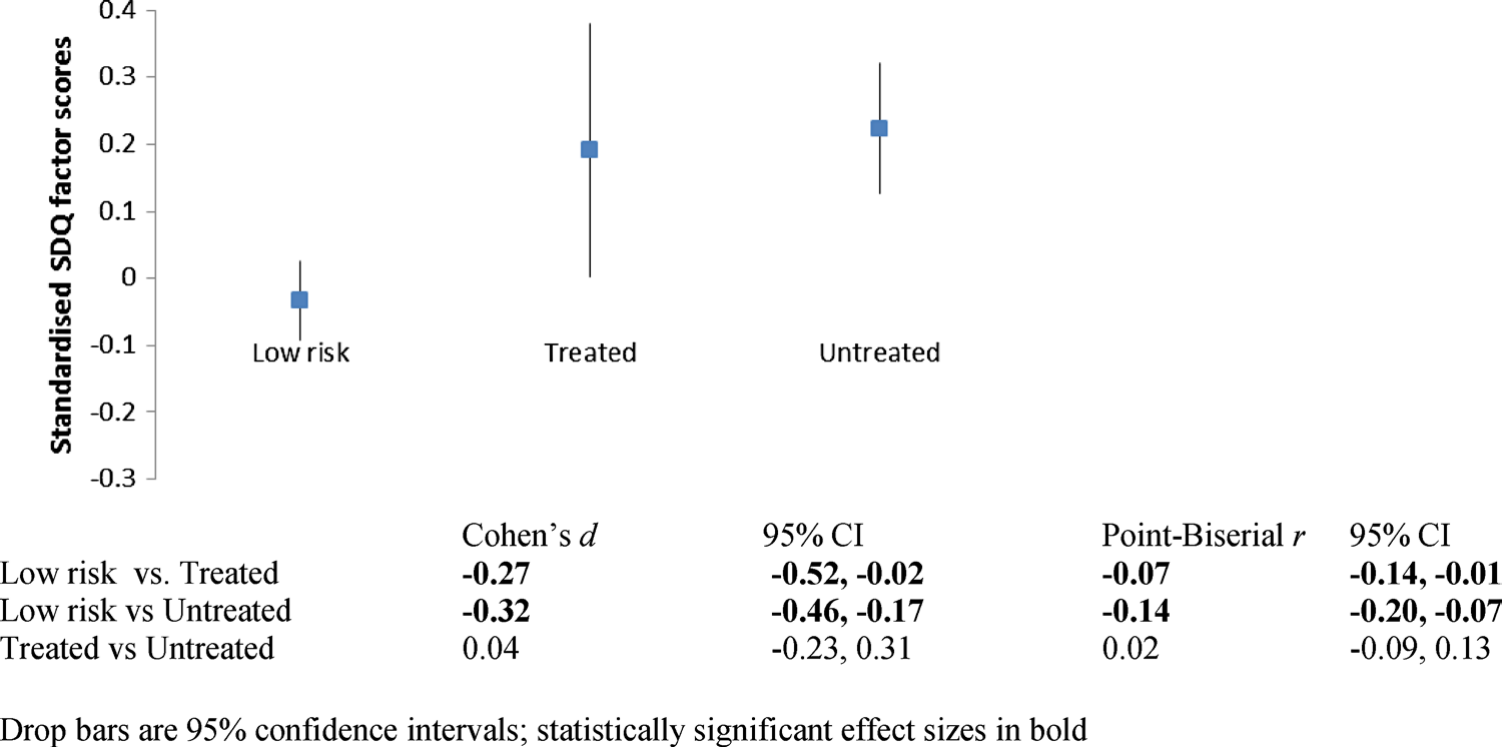
Standardised SDQ factor scores at age three by latent classification of maternal mental health. *Drop bars* are 95 % CI; statistically significant effect sizes in *bold*

The standardised SDQ factor scores for each latent classification with estimates of effect size are presented in Fig. 2. Compared to children of mothers unlikely to have CMD, children with mothers classified as having untreated CMD had higher SDQ factor scores (*d* = 0.32), as did children with mothers classified as having treated CMD (*d* = 0.27). There was little variation in SDQ factor scores between children of mothers with treated and untreated CMD.

The findings were largely unchanged when, adjusting for the sex of the child and ethnic group, the SDQ factor scores for children of women in the treated and untreated groups were contrasted against those whose mothers did not have CMD, and when analysing responses to the single question about perceived behavioural difficulties (Table 4). Further adjusting for a marker of socio-economic difficulties (the number of items lacked on the FRS) resulted in attenuated estimates, but the association between increased child difficulties in offspring of mothers with untreated CMD, compared to mothers unlikely to have CMD, was still visible.

**Table 4:** Linear and poisson multivariate regression of SDQ on latent maternal mental health classification

	*N* (%)	Standardised SDQ factor score^a^			Perceived behavioural difficulties rating^b^		
		Model 1	Model 2	Model 3	Model 1	Model 2	Model 3
		Coeff (95 % CI)	Coeff (95 % CI)	Coeff (95 % CI)	RRR (95 % CI)	RRR (95 % CI)	RRR (95 % CI)
Maternal mental health classification							
Low risk	756 (70.1)	0	0	0	1	1	
Untreated CMD	255 (23.7)	**0.25 (0.14**, **0.37)**	**0.25 (0.14**, **0.37)**	**0.21 (0.09**, **0.32)**	**1.59 (1.13**, **2.23)**	**1.59 (1.13**, **2.23)**	**1.58 (1.13**, **2.23)**
Treated CMD	67 (6.2)	**0.21 (0.02**, **0.40)**	**0.21 (0.02**, **0.41)**	0.17 (−0.02, 0.37)	1.59 (0.93, 2.74)	1.54 (0.90, 2.66)	1.56 (0.90, 2.68)
Child sex							
Boy	514 (47.7)	0	0	0	0	0	0
Girl	564 (52.3)	**−0.16 (−0.26**, **−0.07)**	**−0.16 (−0.26**, **−0.06)**	**−** **0.17** **(** **−** **0.27** **,** **−** **0.07** **)**	**0.62 (0.45**, **0.86)**	**0.62 (0.45**, **0.85)**	**0.62 (0.45**, **0.85)**
Mothers ethnic group							
White British	396 (36.7)	**–**	0	0	–	0	0
Not White British	682 (63.3)	**–**	0.02 (**−**0.06, 0.09)	0.01 (**−**0.06, 0.09)	**–**	0.93 (0.74, 1.16)	0.93 (0.74, 1.16)
Materially deprived	617 (56.9)^c^	**–**	–	**0.10 (0.05**, **0.15)**	**–**	–	1.03 (0.89, 1.20)

Standardised SDQ factor scores were generated by estimating the loading for each child on the 20 item Total Difficulties factor while partitioning out error variance from the internalising, externalising and positively worded questions in a bifactor model; bolded estimates are statistically significant

*Coeff* beta coefficient, *RRR* relative risk ratio

^a^Linear regression

^b^Poisson regression

^c^One or more item lacked on the Family Resources Survey

## Discussion

Using latent class analysis in a cohort linking GP records and research data we identified a substantial group of mothers with a high likelihood of potentially persistent CMD with no record of treatment in primary care. Compared to children of women who were unlikely to have CMD, the 3-year-old children of women with untreated CMD were more likely to be rated as having socio-emotional and behavioural difficulties. Little difference was observed between these difficulties in children whose mothers were treated and those whose mothers were not.

### Strengths and limitations

Women enrolled in BiB1000 were broadly representative of the maternal population of Bradford at the time of recruitment, and although there were some demographic differences between our analysed sample and those excluded due to missing data, there appeared to be little variation in mental health between analysed and excluded women, or indeed in their child’s pattern of behaviour. Therefore, we do not think that missing self-reported data have significantly affected our results or distorted our conclusions. The quantity of missing data from the primary care dataset, however, is unknown; for example CMD treatment noted in a free-text field or a referral letter (only) was not picked up. The tight geographic focus and relatively short (9 months) recruitment period are strengths in that they minimise potential regional and temporal variation in GP coding practice. However, while Bradford may be representative of other ethnically and socio-economically diverse UK cities, our findings may have limited generalisability to other settings.

Our approach sought to minimise the effect of cultural variation in the self-reporting of distress/difficulties by the mother, both for herself and her child. Although numbers were too small in this study to explore differences in latent classification by ethnicity, the association between latent classification and SDQ outcomes appeared robust to variation by ethnic group. We use the term ‘untreated’ as a label for the group of women classified as having an increased likelihood of self-reported distress scores over the 75th centile across three time points without visible note of treatment in the electronic primary care record, and not as an established clinical diagnosis. Our findings may be distorted if we misclassified women who were actually treated for CMD as ‘untreated’, for example if their treatment was noted in a free-text field or letter to which we did not have access, or if the methods we used tended to incorrectly classify women with transient non-pathological distress, or treatment in one particular period, as having untreated CMD (false-positives). Due to small numbers we did not distinguish between types of treatment, and we were unable to distinguish between offer and uptake of treatment, or between failed and successful treatment, which may have introduced heterogeneity in regards to the children’s outcomes. Compromised precision in our analysis for disorder and treatment classification might be a factor in the observed lack of difference between children’s outcomes by maternal treatment status.

Common mental disorders are chronic, relapsing conditions, including over the maternal period [37–39]. This was one of the reasons we chose to model chronicity of mother’s mental health rather than permitting classification on discrete risks in each period. We did not, therefore, capture any dynamic or transitional variation over time, for example trying to characterise women who had either a prenatal or postnatal CMD episode, but not both. That we classified nearly five times more women as ‘untreated’ than ‘treated’ indicates our results may have been in favour of classifying women as ‘untreated’ rather than as ‘treated’. A feature of latent class analysis is that it allows for sample-specific flexibility in modelling approach; some studies using latent class methods to model both trajectory and severity of self-reported symptoms over the maternal period have found that their data were best characterised by periods of discrete risk [40], but others have reported that chronicity models best fit their data [5, 7, 41]. The main driver for predefining a chronicity model was to provide a platform against which we could distinguish group membership based on treatment of CMD in primary care. This approach has merit as a method for estimating risks given that CMDs tend to be chronic and relapse rates are high, the outcomes of treatment in any maternal period are not well studied and effects are modest at best [42–44]. In using a chronicity model and a centile-based threshold approach for the self-reported measures, however, we did not account for potential variation in severity of disorder, which may have implications for treatment offered, or take up [45], or effect on child outcomes. Variation in categorised distress could have also been introduced by alternation of ascertainment by GHQ-28 and Kessler-6 at follow-up, which was beyond our control, although both assess symptoms of psychological distress and our use of a centile threshold may have helped to minimise any effect. Due to small numbers of treated women and uncertainty about timing of incident diagnosis and continuity of treatment events we were unable to unpick whether treatment was sufficient to be effective. Future studies with data linkage for greater temporal ranges and in larger populations are required to answer specific questions on the effectiveness of specific treatment regimes.

We employed a robust psychometric approach to analysing SDQ data, generating scores for each child relative to other children in the sample that control for measurement error through use of an appropriate latent variable model approach. Contrary to other findings in pre-school community samples [20, 46], we were unable to establish an acceptable baseline psychometric model for the theorised structure of the SDQ in this multi-ethnic sample and suggest further validation studies to establish population-based concepts of problem behaviour in such young children. We replicated the factor-score regression findings using the single-item Perceived Difficulties question, but this item has been shown to discriminate between clinical and community samples using the ‘severe’ and ‘serious’ categories [24], not as we used it, within a community sample including ‘minor difficulties’. As with all studies that ask parents to assess their child’s behaviour, our results may be distorted by the effect of more distressed mothers being potentially more likely to rate their child’s behaviour as problematic [47–49]. Future linkage of this sample with routinely collected school attainment data and teacher-rated behaviour may help overcome some of these measurement limitations and establish whether our findings related to untreated CMD are confirmed by more objectively measured outcomes.

### Research findings in context

To the best of our knowledge, this study is the first to attempt to quantify the association between unrecognised maternal mental health problems and child behaviour outcomes, and we found a small, but significant association. Although previous research has found increased levels of child behavioural problems associated with maternal mental health difficulties [1–5, 7, 50], these studies have not accounted for treatment of disorder, which makes direct comparison with our study difficult. We can, however, use data from these studies to help interpret our findings. Goodman et al. [51] estimated a weighted *r* of 0.24 (95 % CI; 0.22, 0.26) in a meta-analysis of 39 observational studies reporting the association between maternal depression and children’s general psychopathology (grand mean age 7). An older review estimated a weighted *r* of 0.26 and weighted *d* of 0.53 for the association between depressed mothers (excluding studies of postnatal depression) and behaviour problems in pre-school children from 11 studies [52]. These average effects are ‘moderate’ in size [53], and are larger than the difference in SDQ scores in our study between children of mothers without CMD and those in either the treated (*d* = 0.27, *r* = 0.07) or untreated (*d* = 0.32, *r* = 0.14) groups. This is particularly noteworthy, as effect sizes have been reported to be larger in studies of low income families and where the children were assessed by their mothers, as in BiB [51]. Aside from differences in setting and population, there are several potential explanations for the smaller observed effects in our study.

First, we categorised women in the ‘no CMD’ group as unlikely to have persistent CMD; however, this group may have contained some women with a disorder (treated or untreated) in a single, potentially sensitive, period. If this were the case, affected children may have skewed the SDQ scores of the low-risk group upwards, reducing the observed effect size relative to studies that screened or diagnosed to classify maternal disorder. Second, children of the treated mothers in BiB may have less psychopathology due to successful maternal treatment of CMD. A meta-analysis of mental health outcomes in infants and very young children after maternal psychological treatment for depression in five small trials found a small to moderate pooled effect size (*g*, broadly equivalent to *d*) of 0.40 (0.21, 0.59) between the treated and control groups [11], indicating that, although long-term effects are unclear, maternal treatments have the potential to have positive effects for children in the short term. If the SDQ scores of BiB children have been moderated by maternal treatment, i.e. their SDQ scores would have been higher had the mothers not been treated, then this raises another question; why are not the SDQ scores in the untreated group higher than the treated group? A plausible explanation is that untreated women had less severe symptoms with less impact on their children. While this might explain the difference in magnitude of our results compared to other studies, even low level or subclinical maternal symptoms can have observable relationships with children’s outcomes [5, 7]. A third explanation for the smaller effects observed in our study is that women who were distressed (in the treated and/or untreated groups) rated their children as having fewer problems on the SDQ for actual level of child problems, relative to mothers without CMD. We think this unlikely as maternal distress has generally been associated with higher child problematic behaviour ratings [47, 48]; however, differential effects may have distorted our findings [49]. Fourth, we examined treatment for anxiety, depression, mixed disorders and symptomology, not just depression, which was the subject of the meta-analyses. As with depression, anxiety disorders have detrimental effects on children [54]; however, differential effects have been noted which may have affected our results if women with one or other of these disorders were clustered in the treated or untreated groups [55].

Much of our health, and mental health, is socially patterned and influenced [56, 57], and previous analyses of the larger BiB cohort and other research datasets confirm that women living in socio-economic disadvantage are more likely to be distressed, or potentially unidentified as being distressed [10, 30, 41, 58]. Severity of child behaviour problems similarly appears to follow socio-economic gradients, whether rated by parents or teachers [59]. We found that a measure of socio-economic disadvantage attenuated but did not remove the increased likelihood of higher SDQ scores in children of women with untreated CMD compared to scores of children whose mothers did not have CMD. This indicates that our findings are not confounded by socio-economic status, although residual confounding by ethnicity is a possibility that we could not explore due to small sample size. In practice, social and economic disadvantage are likely to exert rather complex and variable effects on whether a women with CMD is identified as such, and mitigate or exacerbate subsequent effects of distress on her family.

### Implications for research and policy

Our study is unique in that we attempted to estimate the magnitude of behaviour problems displayed by children of women who are likely to be potentially unidentified as distressed and, therefore, untreated, by the health service. The size of the association did not appear to vary between treated and untreated women. As discussed above, this could be interpreted, broadly, as indicating that currently offered treatment in this cohort might not be successful in mitigating the effects of maternal anxiety and depression on children, or that currently offered treatment has mediated but not remedied the effect on children, or that untreated women have less severe symptoms which have less impact on their children.

There is thus far only a limited evidence base for treatment effectiveness in trials examining outcomes on mothers [42–44], and even less for trials examining the effect of maternal, partnership or family treatment on children’s outcomes [11, 60]. Robust trials that test interventions to improve mother and baby interaction and responsiveness as early markers of difficulties with longitudinal follow-up are needed with accurate assessment of the interaction problem with targeted intervention [45, 61]. Studies that examine the specific effects of anxiety and/or depression on children’s functioning and behaviours are also needed. We suggest that the development of treatment innovation and improved access to psychological therapy should be complemented with more effort and resource applied to identify anxiety and depression in maternal women as a precursor to getting them into treatment, and the implementation of any successful treatment programme into routine health services should be subject to a rigorous inequality evaluation.

In previous analyses of the larger BiB cohort, we have noted ethnic disparities in both the identification and treatment of maternal CMD [10, 62], of which the absolute and relative distal effects on children need to be explored in a larger dataset. A greater understanding of why some women who are experiencing persistent difficulties are not identified as such in primary care is needed.

It is possible, and likely, that in our study, women in the untreated group had less severe symptoms or transient distress that may not meet current criteria for treatment. If this is the case, our study confirms previously reported research about the effect of sub-threshold disorder and persistent low-level symptoms on children’s outcomes [5, 7]. More research attention is needed in this area such as trials of treatment for women with less severe symptoms that include long-term follow-up of their children.

### Implications for practice

It is now widely accepted that, in addition to depressive problems, anxiety disorders in the maternal period cause significant morbidity [45, 63, 64]. Recently updated UK guidance for managing maternal mental health advises clinicians to consider screening for anxiety as well as depression at each contact, consider the needs of vulnerable women, and to be aware that some women may be unwilling to disclose their distress [45]. Treatment staging advice and maximum time-to-treatment targets are clearly specified [45]. There is, however, little focused advice on how to ensure that screening for any CMD is as successful in identifying cases among disadvantaged groups, or ethnic minority women, as more advantaged, or majority populations, or ensuring equitable treatment outcomes. Clinical reality in overstretched health services means that screening, referrals and treatment availability for mental health in maternal or primary care may be sub-optimal, highlighting the gap between evidence-based advice and practice [65]. Primary and community care staff need help to identify patients who may not, on first appearances, appear to be vulnerable, to translate population or community-level socio-demographic information into individual risk assessments for poor outcomes, and to understand the impact of health inequalities on their patients [66].

## Conclusions

In this multi-ethnic city-based birth cohort the 3-year-old offspring of women who potentially have untreated anxiety and depression are at higher risk for socio-emotional and behavioural problems than the children of unaffected women, and have an equally elevated risk compared to children of mothers treated for their disorder. The development of effective treatments for CMD needs to be balanced by greater attempts to identify and treat women.

## Electronic supplementary material

Below is the link to the electronic supplementary material.

Supplementary material 1 (DOCX 28 kb)
