# Supplementary material for: Maternal psychological distress in primary care and association with child behavioural outcomes at age three
Source: Eur Child Adolesc Psychiatry. 2015 Sep 28;25:601–13. doi: 10.1007/s00787-015-0777-2 (PMC4889639; doi:10.1007/s00787-015-0777-2)
Supplement: Supplementary file 1 — Supplementary material 1 (DOCX 28 kb) [file 787_2015_777_MOESM1_ESM.docx]

**Supplementary online appendix**

**Table S1. List of drug prescriptions and Read codes**

| Indication | Classification / action | List |
| --- | --- | --- |
| **Drugs** |  |  |
| Drugs used to treat CMD | Treated | agomelatine, alprazolam, alventa, alventa xl, angilol, ativan, bonilux, bonilux xl, buspironehydrochloride, chloralbetaine, chloralhydrate, chloralmixture, bp2000, cipralex, cipramil, circadin, citalopram, clomipramine, clomipraminehydrochloride, clonezapam, depefex, depefex xl, diazepam, dosulepin, dosulepinhydrochloride, edronax, efexor, efexor xl, escitalopram, faverinparoxetine, feprapax, fluoxetine, flurazepam, fluvoxamine, fluvoxaminemaleate, foraven, foraven xl, gamanil, hloralhydrate, imipramine, imipraminehydrochloride, isocarboxazid, lofepramine, lomont, loprazolam, lorazepam, lormetazepam, lustral, manerix, marplan, melatonin, meprobamatemeprobamate, mianserin, mianserinhydrochloride, mirtazapine, moclobemide, molipaxin, nardil, nitrazepam, optimax, oxactin, oxazepam, parnate, paroxetine, phenelzine, politid, politid xl, propranolol, propranololhydrochloride, prothiaden, prozac, ranfaxine, ranfaxine xl, reboxetine, seroxat, sertraline, sonata, stilnoct, surmontil, syprol, temazepam, tifaxin, tifaxin xl, tranylcypromine, trazodone, trazodonehydrochloride, trimipramine, tryptophan, valdoxan, venaxx, venaxx xl, venlafaxine, venlafaxine m/r, vensir, vensir xl, welldorm, winfex, winfex xl, zaleplon, zimovane, zispin, zispinsoltab, zolpidem, zolpidemtartrate, zopiclone. |
| Drugs used to treat SMI | Cases dropped | abilify, acuphase, amisulpride, anquil, aripiprazole, asenapine, benperidol, camcolit, clopixol, clopixol, clozapine, clozaril, denzapine, depixol, depixol conc., depixol low volume, dolmatil, fluanxol, flupentixol, flupentixol decanoate, fluphenazine decanoate, haldol decanoate, invega, li-liquid, liskonum, lithium carbonate, lithium citrate, modecate, modecate concentrate, olanzapine, olanzapine embonate, orap, paliperidone, pericyazine, pimozide, piportil depot, pipotiazine,, palmitate, priadel, promazine, promazine hydrochloride, quetiapine, risperdal, risperdal consta, risperidone, seroquel, seroquel xl, solian, sulpiride, sulpor, sycrest, xeplion, zaponex, zuclopenthixol, zuclopenthixol acetate, zuclopenthixol decanoate, zypadhera, zyprexa. |
| Drugs used to treat SMI and non-mental health conditions | Cases dropped | carbamazepine, chlorpromazine, chlorpromazine hydrochloride , convulex, depakote, dozic, fentazin, haldol, haloperidol, largactil, levomepromazine, nozinan, perphenazine, serenace, stelazine, tegretol trifluoperazine, valproic acid. |
| **Read codes** |  |  |
| CMD treatment or referral for CMD treatment | Treated | 6655., 6659., 66590, 6779., 6G00., 8BK0., 8BM0., 8CQ.., 8CR7., 8F85., 8G..., 8G1.., 8G10., 8G100, 8G11., 8G12., 8G120, 8G121, 8G2.., 8G21., 8G2Z., 8G4.., 8G43., 8G4Z., 8G5.., 8G51., 8G5Z., 8G6.., 8G6Z., 8G7.., 8G7Z., 8G9.., 8G91., 8G9Z., 8HlB., 8HVO., 8H23., 8H230, 8H34., 8H38., 8H49., 8H7A., 8H7B., 8H7T., 8H7Z., 8HHp., 8HHq., 8HJ3., 8HK9., 8HkK., 8HM9., 9HZ.., 9N0T., 9N1M., 9N2B., 9N6h., 9NJ1., 9NJR., 9NJT., 9Ol.., Ub0qs, X71Ec, X71bp, X79sL, XE0iL, XE1Sa, XE1Sb, XSBbs, Xa8IB, Xa8IG, Xa8IJ, Xa8IP, Xa8IR, Xa8If, Xa8Ig, Xa8Ih, Xa8Ii, Xa8Ij, Xa8Ik, Xa8Is, Xa8It, Xa8Iu, Xa8Ix, Xa8J0, XaA8Z, XaA8c, XaA8d, XaA8u, XaA8v, XaA9W, XaA9g, XaABP, XaABQ, XaAKy, XaAMj, XaAMz, XaAOd, XaAOe, XaAOf, XaAOg, XaAOh, XaAQi, XaAQo, XaAS4, XaAU5, XaAUA, XaAXe, XaAZI, XaAbC, XaAbH, XaAdM, XaAel, XaAem, XaAen, XaAfJ, XaAh4, XaAiE, XaAiI, XaAkB, XaAkI, XaAkU, XaAnb, XaBHK, XaBIg, XaBJb, XaBJc, XaBT1, XaBTD, XaBtN, XaBvV, XaBvW, XaBvX, XaCFD, XaECG, XaEVq, XaI8j, XaINQ, XaINy, XaIOf, XaIOg, XaIOh, XaIOi, XaIOj, XaIOk, XaIOl, XaIOn, XaIOp, XaIOq, XaIOs, XaIOu, XaIOv, XaIOy, XaIOz, XaIP0, XaIP1, XaIP2, XaIP3, XaIPw, XaISp, XaISv, XaISw, XaISy, XaIT1, XaIT2, XaIT3, XaIT4, XaIT5, XaIT6, XaIT7, XaIT8, XaITA, XaITG, XaITH, XaITI, XaIUv, XaIUx, XaIUy, XaIUz, XaIV0, XaIV1, XaIV2, XaIV3, XaIV4, XaIV5, XaIV6, XaIW3, XaIW4, XaIW5, XaIW6, XaIWD, XaIWM, XaIWN, XaIWR, XaIWS, XaIWT, XaIWU, XaIWV, XaIWW, XaIWX, XaIWY, XaIWZ, XaIWa, XaIWb, XaIWx, XaIWy, XaIWz, XaIX0, XaIXS, XaIXT, XaIXU, XaIXV, XaIXW, XaIXX, XaIXY, XaIXZ, XaIXa, XaIXb, XaIXh, XaIXi, XaIXk, XaIXl, XaIXm, XaIXn, XaIXo, XaIXp, XaIXq, XaIXs, XaIXt, XaIXu, XaIYN, XaIkd, XaIkg, XaIku, XaIm4, XaIpA, XaItc, XaItx, XaIuR, XaIvk, XaIvp, XaIvq, XaIyU, XaJ4V, XaJ4w, XaJ4x, XaJOA, XaJON, XaJPu, XaJPz, XaJQ1, XaJQD, XaJQE, XaJQF, XaJQG, XaJQH, XaJQI, XaJQJ, XaJQR, XaJQS, XaJQT, XaJQU, XaJQV, XaJQW, XaJQX, XaJQY, XaJQZ, XaJRr, XaJWg, XaJr3, XaK1f, XaK5q, XaK5r, XaK6K, XaK70, XaK71, XaKAx, XaKEz, XaKGq, XaKbb, XaL03, XaL0o, XaL0p, XaL0q, XaL0r, XaL0s, XaL0t, XaL0u, XaL0v, XaL0w, XaL2L, XaLBl, XaLCP, XaLCQ, XaLFL, XaLFk, XaLNF, XaLQw, XaLnp, XaLnq, XaLnr, XaLst, XaLsu, XaLsv, XaM2K, XaM7s, XaMGz, XaMJ8, XaMhM, XaN3a, XaN4b, XaN4c, XaN4d, XaN4e, XaN4f, XaN4g, XaNPL, XaNTc, XaONq, XaOOT, XaObo, XaOxM, XaP6T, XaP7x, XaPRF, XaPTT, XaPTU, XaPlZ, XaPvy, XaPvw, XaQBz, XaQC0, XaQWJ, XaQvz, XaR4n, XaR4s, XaR5D, XaWzW, XaX04, XaXEJ, XaXH8, XaXHm, XaXe3, XaXiH, XaXl2, XaY6o, XaY7i, XaYgS, XaZIW, XaZcf, ZV663, ZV673, ZV69., ZV690, ZV691, ZV692, ZV6D., ZV701, ZV702 |
| CMD-related follow-up | Treated | 665.., 6654., 6658., 66580, 665A., 665A0, 665Z., 8A2.., 8A21., 8A2Z., 9H90., 9H91., 9H92., 9HA0., 9Ov.., 9Ov0., 9Ov1., 9Ov2., 9Ov3., 9Ov4., X74WN, XaJuG, XaJuK, XaJuT, XaJuV, XaJuW, XaK6d, XaK6e, XaK6f, XaK9p, XaKAK, XaLIb, XaMGL, XaMGN, XaMGO, XaMGP, XaMGQ, XaMGR, XaR9y, XaZ2p |
| SMI | Cases dropped | 1B1b., 225E., 225F., 6656., 6657., 665B., 665C., 665D., 665E., 665F., 665G., 665H., 665J., 665K., 8HHs., 9H1.., 9H11., 9H12., 9H13., 9H14., 9H1Z., 9H2.., 9H21., 9H22., 9H23., 9H24., 9H25., 9H2Z., 9H3.., 9H31., 9H32., 9H33., 9H34., 9H3Z., 9H4.., 9H41., 9H42., 9H43., 9H44., 9H45., 9H4Z., 9H5.., 9H51., 9H52., 9H53., 9H54., 9H55., 9H5Z., 9H7.., 9H8.., 9Ol6., 9Ol7., E10.., E100., E1000, E1001, E1002, E1003, E1004, E1005, E100z, E101., E1010, E1011, E1012, E1013, E1014, E1015, E101z, E102., E1020, E1021, E1022, E1023, E1024, E1025, E102z, E103., E1030, E1031, E1032, E1033, E1034, E1035, E103z, E104., E110., E1100, E1101, E1102, E1103, E1104, E1105, E1106, E110z, E111., E1110, E1111, E1112, E1113, E1114, E1115, E1116, E111z, E1124, E1133, E1134, E114., E1140, E1141, E1142, E1143, E1144, E1145, E1146, E114z, E115., E1150, E1151, E1152, E1153, E1154, E1155, E1156, E115z, E116., E1160, E1161, E1162, E1163, E1164, E1165, E1166, E116z, E117., E1170, E1171, E1172, E1173, E1174, E1175, E1176, E117z, E11y., E11y0, E11y1, E11y3, E11yz, E11z., E12.., E120., E121., E122., E123., E12y., E12y0, E12yz, E12z., E13.., E130., E131., E132., E133., E134., E135., E13y., E13y0, E13y1, E13yz, E13z., E14.., E141., E1411, E141z, E14y., E14y1, E14yz, E14z., E1y.., E1z.., E21.., E2111, E2112, E2113, E211z, E212., E2120, E2121, E2122, E212z, E213., E214., E2140, E2141, E214z, E215., E2150, E2151, E2152, E2153, E215z, E216., E217., E21y., E21y1, E21y2, E21y3, E21y4, E21y5, E21y6, E21y7, E21yz, E21z., Eu1.., Eu10., Eu100, Eu101, Eu102, Eu103, Eu104, Eu105, Eu106, Eu107, Eu108, Eu10y, Eu10z, Eu11., Eu110, Eu111, Eu112, Eu113, Eu114, Eu115, Eu116, Eu117, Eu11y, Eu11z, Eu12., Eu120, Eu121, Eu122, Eu123, Eu124, Eu125, Eu126, Eu127, Eu12y, Eu12z, Eu13., Eu130, Eu131, Eu132, Eu133, Eu134, Eu135, Eu136, Eu137, Eu13y, Eu13z, Eu14., Eu140, Eu141, Eu142, Eu143, Eu144, Eu145, Eu146, Eu147, Eu14y, Eu14z, Eu15., Eu150, Eu151, Eu152, Eu153, Eu154, Eu155, Eu156, Eu157, Eu15y, Eu15z, Eu16., Eu160, Eu161, Eu162, Eu163, Eu164, Eu165, Eu166, Eu167, Eu16y, Eu16z, Eu17., Eu170, Eu171, Eu172, Eu173, Eu174, Eu175, Eu176, Eu177, Eu17y, Eu17z, Eu18., Eu180, Eu181, Eu182, Eu183, Eu184, Eu185, Eu186, Eu187, Eu18y, Eu18z, Eu19., Eu190, Eu191, Eu192, Eu193, Eu194, Eu195, Eu196, Eu197, Eu19y, Eu19z, Eu1A., Eu1A0, Eu1A1, Eu1A2, Eu1A3, Eu1A4, Eu1A5, Eu1A6, Eu1A7, Eu1Ay, Eu1Az, Eu2.., Eu20., Eu200, Eu201, Eu202, Eu203, Eu204, Eu205, Eu206, Eu20y, Eu20z, Eu21., Eu22., Eu220, Eu221, Eu222, Eu223, Eu22y, Eu22z, Eu23., Eu230, Eu231, Eu232, Eu233, Eu23y, Eu23z, Eu24., Eu25., Eu250, Eu251, Eu252, Eu25y, Eu25z, Eu26., Eu2y., Eu2z., Eu30., Eu300, Eu301, Eu302, Eu30y, Eu30z, Eu31., Eu310, Eu311, Eu312, Eu313, Eu314, Eu315, Eu316, Eu317, Eu318, Eu319, Eu31y, Eu31z, Eu323, Eu328, Eu329, Eu32A, Eu333, Eu341, Eu3z., Eu44., Eu440, Eu441, Eu442, Eu443, Eu444, Eu445, Eu446, Eu447, Eu44y, Eu44z, Eu45., Eu450, Eu451, Eu452, Eu453, Eu454, Eu455, Eu45y, Eu45z, Eu46., Eu460, Eu461, Eu46y, Eu46z, Eu5.., Eu531, Eu54., Eu55., Eu5z., Eu6.., Eu61., Eu60., Eu601, Eu602, Eu603, Eu604, Eu605, Eu606, Eu607, Eu608, Eu60y, Eu60z, Eu62., Eu620, Eu621, Eu62y, Eu62z, Ua1WW, Ub1T7, X73gl, X73gm, X73gn, X73go, X75yp, X75yv, X75yw, X75z5, X75z7, X75zA, X75zC, X75zE, X761M, X79ul, XE1Xt, XE1Xw, XE1Xx, XE1Y2, XE1Y3, XE1Y4, XE1Y5, XE1Y6, XE1YF, XE1YG, XE1YH, XE1YI, XE1YJ, XE1YK, XE1YL, XE1YM, XE1ZM, XE1ZN, XE1ZO, XE1ZP, XE1ZQ, XE1ZR, XE1ZU, XE1ZX, XE1ZZ, XE1Ze, XE1Zy, XE1aM, XE1aO, XE1aQ, XE1aS, XE1aU, XE1ag, XE1am, XE1gG, XE1ic, XE1ji, XE2RN, XE2b6, XE2b8, XE2uT, XE2un, XE2v2, XM1GG, XM1Yd, XSGon, Xa1aD, Xa1hV, Xa3WO, Xa3Xd, Xa3Xf, Xa3Xg, Xa3Y9, Xa3aC, Xa3aF, Xa3aL, Xa3aP, Xa3aU, Xa3aV, Xa3aW, Xa3aX, Xa3at, Xa4HV, Xa4Ha, Xa8Nf, Xa8O2, Xa8OA, Xa8OE, Xa8Og, Xa8Oh, Xa8Oi, Xa8Pk, Xa8Qw, Xa8Qx, Xa8Qy, Xa8Qz, Xa9Dh, Xa9Di, Xa9Dk, Xa9Dm, Xa9Do, Xa9Dr, Xa9Ds, Xa9Dt, Xa9Du, Xa9Dv, Xa9Dw, Xa9Dx, Xa9Dy, Xa9Dz, Xa9E1, Xa9E2, Xa9EC, Xa9EE, Xa9EF, Xa9EG, Xa9EI, Xa9EM, Xa9EP, Xa9EQ, Xa9ER, Xa9EV, Xa9EW, Xa9EX, Xa9EY, Xa9EZ, Xa9Ea, Xa9Eb, Xa9Ec, Xa9Ed, Xa9Ee, Xa9Ef, Xa9Eg, Xa9Ei, Xa9Ej, Xa9Ek, Xa9El, Xa9Em, Xa9Eo, Xa9Ep, Xa9Eq, Xa9Er, Xa9Es, Xa9Et, Xa9Eu, Xa9Ev, Xa9GR, Xa9GU, Xa9GV, Xa9GX, Xa9GZ, Xa9Gc, Xa9IW, Xa9IX, Xa9IY, Xa9IZ, Xa9Ib, Xa9Ic, Xa9Id, Xa9Ie, Xa9If, Xa9Ig, Xa9Ih, Xa9Ii, Xa9Ij, Xa9Ik, Xa9Il, Xa9Im, Xa9In, Xa9Ip, Xa9Iq, Xa9Is, Xa9Iw, Xa9Iy, Xa9J3, Xa9J4, Xa9J5, Xa9J6, Xa9JA, Xa9JC, Xa9JE, Xa9JG, Xa9JH, Xa9JJ, Xa9JK, Xa9JR, Xa9Ja, Xa9Jb, Xa9Jd, Xa9Jf, Xa9Jh, Xa9Jj, Xa9Jl, Xa9Jn, Xa9Jp, Xa9Jq, Xa9Jr, Xa9Js, Xa9Jt, Xa9Jz, Xa9K2, Xa9K3, Xa9K4, Xa9K5, XaA6j, XaA6x, XaA9j, XaA9r, XaA9s, XaBHL, XaBHM, XaBHN, XaBHO, XaBYV, XaBYW, XaBYX, XaBYY, XaBYZ, XaBhM, XaCHo, XaIOm, XaIWE, XaIWF, XaIWG, XaIWH, XaIWI, XaIWJ, XaIWK, XaIWL, XaIXj, XaJQO, XaKUl, XaKUm, XaL19, XaLIa, XaMwc, XaMwd, XaMwe, XaNlN, XaPYK, XaPYL, XaX51, XaX52, XaX53, XaX54, XaY1Y |

Case-free searching employed for drugs; CMD common mental disorder; SMI severe mental illness

**Table S2. Descriptive characteristics of the included and excluded sample**

|  | Analysed | Excluded (1) | Excluded (2) | Excluded combined (1) & (2) | Analysed vs. excluded combined Chi^2^(df), P |
| --- | --- | --- | --- | --- | --- |
| N | 1,078 | 335 | 322 | 657 |  |
| Ethnic group (language), N (%) |  |  |  |  |  |
| White British | 396 (36.7) | 140 (41.8) | 124 (38.5) | 264 (40.1) | 15.1(3)  P=0.002 |
| Pakistani (English) | 329 (30.5) | 83 (24.8) | 101 (31.4) | 184 (28.0) |  |
| Other (English) | 123 (11.4) | 71 (21.2) | 34 (10.6) | 105 (16.0) |  |
| Any (Not English) | 230 (21.3) | 41 (12.2) | 63 (19.6) | 104 (15.8) |  |
| Migration history |  |  |  |  |  |
| Born in the UK | 664 (61.6) | 211 (63.0) | 208 (64.6) | 419 (63.8) | 1.1(2)  P=0.57 |
| Migrated to UK before age 16 | 81 (7.5) | 22 (6.6) | 21 (6.5) | 43 (6.5) |  |
| Migrated to UK on or after age 16 | 327 (30.3) | 98 (29.3) | 92 (28.6) | 190 (28.9) |  |
| *missing* | 6 (0.6) | 4 (1.2) | 1 (0.3) | 5 (0.8) |  |
| Age at enrolment, mean (SD) | 27.6 (5.7) | 26.5 (5.5) | 26.5 (5.7) | 26.5 (5.6) | ^b^t=3.9, P<0.001 |
| Relationship status at recruitment N (%) |  |  |  |  |  |
| Married and living together | 760 (70.5) | 212 (63.3) | 203 (63.0) | 415 (63.2) | 14.1(2)  P=0.001 |
| Cohabiting | 181 (16.8) | 72 (21.5) | 48 (14.9) | 120 (18.3) |  |
| Not living with a partner | 133 (12.3) | 51 (15.2) | 70 (21.7) | 121 (18.4) |  |
| *missing* | 4 (0.4) | 0 | 1 (0.3) | 1 (0.2) |  |
| IMD national rank quintile |  |  |  |  |  |
| Most deprived | 718 (66.6) | 231 (69.0) | 225 (69.9) | 456 (69.4) | 3.4(4)  P=0.49 |
| 2 | 206 (19.1) | 51 (15.2) | 58 (18.0) | 109 (16.6) |  |
| 3 | 112 (10.4) | 40 (11.9) | 30 (9.3) | 70 (10.7) |  |
| 4 | 27 (2.5) | 6 (1.8) | 5 (1.6) | 11 (1.7) |  |
| Least deprived | 15 (1.4) | 7 (2.1) | 4 (1.2) | 11 (1.7) |  |
| Number of items lacked on the Family Resources Survey at recruitment | | | | | |
| None | 461 (42.8) | 151 (45.1) | 129 (40.1) | 280 (42.6) | 1.3(3)  P=0.74 |
| 1-2 | 298 (27.6) | 87 (26.0) | 83 (25.8) | 170 (25.9) |  |
| 3-4 | 165 (15.3) | 44 (13.1) | 54 (16.8) | 98 (14.9) |  |
| 5+ | 145 (13.5) | 49 (14.6) | 50 (15.5) | 99 (15.1) |  |
| *missing* | 9 (0.8) | 4 (1.2) | 6 (1.9) | 10 (1.5) |  |
| ≥ 75^th^ centile of self-reported distress measure, N (%)^a,c^ | |  |  |  |  |
| GHQ-28 during pregnancy | 302 (28.1) | 91 (27.5) | 83 (26.1) | 174 (26.8) | 0.35(1) P=0.56 |
| *missing* | 4 (0.4) | 4 (1.2) | 4 (1.2) | 8 (1.2) |  |
| GHQ-28 at 6 months | 257 (28.7) | 68 (34.2) | 52 (27.2) | 120 (30.8) | 0.57(1) P=0.45 |
| *missing* | 182 (16.9) | 136 (40.6) | 131 (40.7) | 267 (40.6) |  |
| Kessler-6 at 12 months | 258 (27.4) | 55 (30.6) | 58 (34.5) | 113 (32.5) | 3.2(1) P=0.07 |
| *missing* | 136 (12.6) | 155 (46.3) | 154 (47.8) | 309 (47.0) |  |
| GHQ-28 at 18 months | 251 (25.8) | 43 (27.4) | 46 (33.8) | 89 (30.4) | 2.3(1) P=0.13 |
| *missing* | 107 (9.9) | 178 (53.1) | 186 (57.8) | 364 (55.4) |  |
| Kessler-6 at 24 months | 248 (25.2) | 42 (32.3) | 28 (29.8) | 70 (31.3) | 3.4(1) P=0.06 |
| *missing* | 94 (8.7) | 205 (61.2) | 228 (70.8) | 433 (65.9) |  |
| Has CMD treatment Read codes / prescriptions, N (%) | |  |  |  |  |
| N | 1,078 | - | 322 | 322 |  |
| Pre-birth | 79 (7.3) | - | 31 (9.7) | 31 (9.7) | 1.8(1) P=0.18 |
| In first postnatal year | 118 (11.0) | - | 40 (12.5) | 40 (12.5) | 0.54(1) P=0.46 |
| In second postnatal year | 139 (12.9) | - | 47 (14.6) | 47 (14.6) | 0.62(1) P=0.43 |
| SDQ Perceived Difficulties question, N (%)^a^ | |  |  |  |  |
| N | 1,078 | 335 | - | 335 |  |
| Severe, serious or minor | 137 (12.7) | 20 (16.8) | - | 20 (16.8) | 1.5(1) P=0.22 |
| *missing* | 0 | 216 (64.5) | - | 216 (64.5) |  |

(1) did not have linked medical records, moved from Bradford, or did not participate in any postnatal sweeps; (2) had linked records, remained in Bradford, missing SDQ; ^a^percentage of non-missing scores; ^b^t-test; ^c^centiles presented in this table were calculated using the whole BiB100 sample (N=1,735); IMD index of multiple deprivation; GHQ General Health Questionnaire; SDQ Strengths and Difficulties Questionnaire.

**Table S3. Latent Class Analysis modelling results**

| Solution | No. free parameters | L^2^ | AIC | BIC | Bootstrapped VLMR LRTest (P) |
| --- | --- | --- | --- | --- | --- |
| 1-class | 6 | -3125.188 | 6262.376 | 6292.273 | - |
| 2-class | 13 | -2919.873 | 5865.747 | 5930.524 | - |
| 3-class | 20 | -2857.761 | 5755.522 | 5855.179 | 2 v 3 p<0.001 |
| 4-class | 27 | -2845.669 | 5745.339 | 5879.876 | 3 v 4 p=0.0128 |
| 5-class | 34 | -2840.779 | 5749.559 | 5918.976 | 4 v 5 p=0.36 |

L^2^, likelihood ratio test; AIC, Akaike’s information criterion; BIC, Bayesian information criterion; VLMR LR, Vuong–Lo–Mendell–Rubin likelihood ratio test.
